# A quantitative and non-contact technique to characterise microstructural variations of skin tissues during photo-damaging process based on Mueller matrix polarimetry

**DOI:** 10.1038/s41598-017-14804-z

**Published:** 2017-10-31

**Authors:** Yang Dong, Honghui He, Wei Sheng, Jian Wu, Hui Ma

**Affiliations:** 1 Shenzhen Key Laboratory for Minimal Invasive Medical Technologies, Institute of Optical Imaging and Sensing, Graduate School at Shenzhen, Tsinghua University, Shenzhen, 518055 China; 2 Department of Biomedical Engineering, Tsinghua University, Beijing, 100084 China; 3 Center for Precision Medicine and Healthcare, Tsinghua-Berkeley Shenzhen Institute, Shenzhen, 518071 China; 4 Department of Physics, Tsinghua University, Beijing, 100084 China

## Abstract

Skin tissue consists of collagen and elastic fibres, which are highly susceptible to damage when exposed to ultraviolet radiation (UVR), leading to skin aging and cancer. However, a lack of non-invasive detection methods makes determining the degree of UVR damage to skin in real time difficult. As one of the fundamental features of light, polarization can be used to develop imaging techniques capable of providing structural information about tissues. In particular, Mueller matrix polarimetry is suitable for detecting changes in collagen and elastic fibres. Here, we demonstrate a novel, quantitative, non-contact and *in situ* technique based on Mueller matrix polarimetry for monitoring the microstructural changes of skin tissues during UVR-induced photo-damaging. We measured the Mueller matrices of nude mouse skin samples, then analysed the transformed parameters to characterise microstructural changes during the skin photo-damaging and self-repairing processes. Comparisons between samples with and without the application of a sunscreen showed that the Mueller matrix-derived parameters are potential indicators for fibrous microstructure in skin tissues. Histological examination and Monte Carlo simulations confirmed the relationship between the Mueller matrix parameters and changes to fibrous structures. This technique paves the way for non-contact evaluation of skin structure in cosmetics and dermatological health.

## Introduction

Skin tissue is comprised of elastic fibres, collagen fibres and matrix composed of protein polysaccharide. It is highly susceptible to damage following long-term exposure to ultraviolet radiation (UVR)^1–3^. Many methods have been developed for the prevention of skin aging and disease caused by daily UVR exposure, such as the use of various kinds of sunscreens. However, because effective ways for non-invasively obtaining structural information about skin tissues are lacking, it is difficult to determine the degree of UVR damage and the effect of sunscreens in real time^4^. Therefore, a non-contact and low-cost technique for quantitatively monitoring the structural variation in skin tissues would be helpful in the fields of both cosmetics and dermatological health. Over the past decades, non-invasive optical techniques such as confocal laser scanning and optical coherence tomography (OCT) have been applied for the imaging of human skin^5,6^. Particularly, many studies have shown that OCT is a promising technique to evaluate the ultraviolet (UV) photo-damage of skin by imaging the epidermis and upper dermis layers *in vivo* ^7^. Using OCT, structural changes and inflammatory reactions induced by UV photo-damage can be revealed non-invasively^8,9^.

Polarization imaging, especially Mueller matrix polarimetry, has many unique advantages as a non-contact and *in situ* technique for detecting tissue microstructures^10–13^. Mueller matrices and Stokes polarimetry can improve the imaging contrast of superficial sample layers and are compatible with other optical systems to expand their ability to acquire microstructural information^14,15^. For example, recent studies demonstrated that polarization-resolved second harmonic generation (SHG) microscopy can be used to determine changes of collagen fibres in different tissues^16,17^. Furthermore, the Mueller matrix can provide a comprehensive characterisation of the polarization-related properties of the sample; hence it has found increasing application in the characterisation of biological tissues^18,19^, textiles^20,21^, plasmonic nanoparticles and other structures^22^. Recently, groups of quantitative parameters with clear physical meanings have been obtained from Mueller matrix elements^23,24^. Preliminary biomedical studies of various pathological tissues, such as colon cancer^25,26^, cervical cancer^27^, skin cancer^28^ and liver fibrosis^29,30^, have shown the diagnostic potential of these Mueller matrix parameters.

Here, we used Mueller matrix polarimetry to characterise the microstructural changes of nude mouse skin during the processes of photo-damaging and self-repairing. We undertook quantitative studies of the structural features of skin tissue through the different stages of these processes by collecting two dimensional (2D) backscattering Mueller matrix images of skin samples at 24 hour intervals, and then calculating the corresponding Mueller matrix transformation (MMT) parameters^24^. The experimental results were compared with Monte Carlo (MC) simulations based on the sphere-cylinder birefringence model (SCBM), which incorporates a birefringent interstitial medium together with spherical and cylindrical scatterers to mimic the organelles and fibres in skin tissues^31–33^. The consistency of the experimental and simulated data demonstrates that the Mueller matrix-derived parameters non-invasively provide indicators for quantitatively monitoring the microstructural features of skin tissues, and may have good prospects for application in the fields of cosmetics and dermatological health.

## Results

### Experimental Setup and Nude Mouse Skin Samples

**Figure 1:**
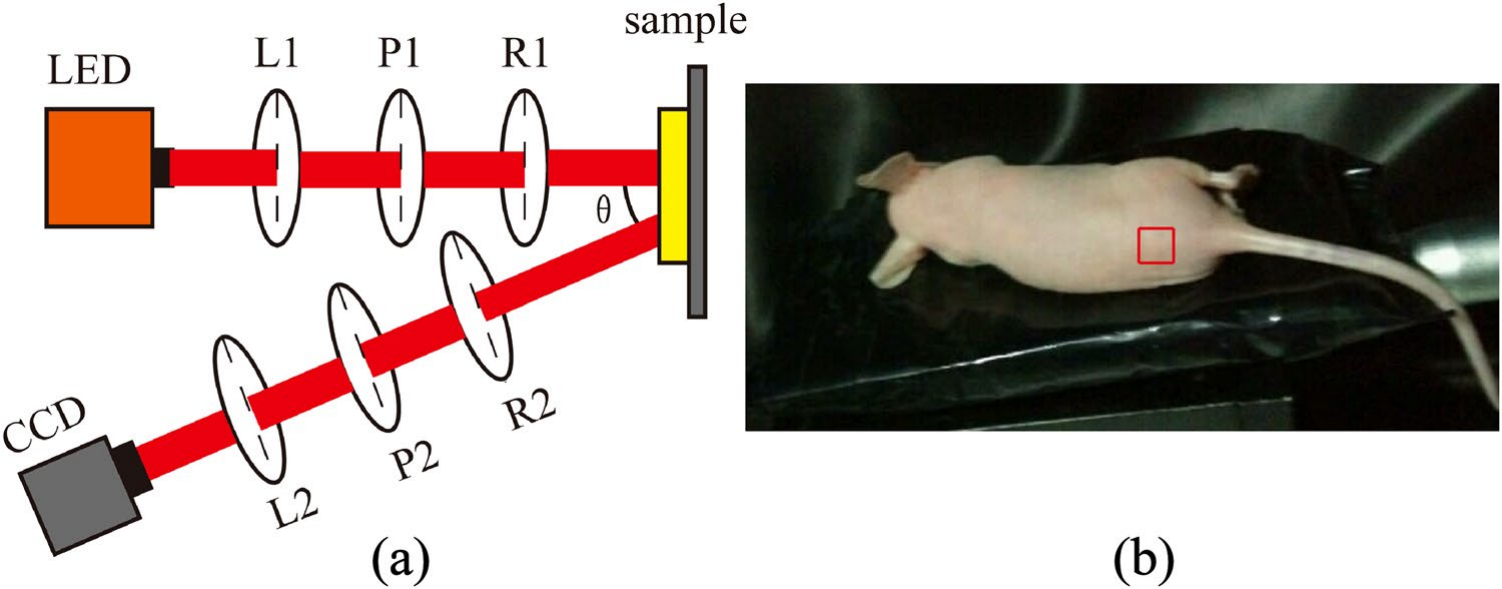
Experimental setup. (**a**) Schematic of the experimental setup for backscattering Mueller matrix measurement. P1, P2: polarizer; R1, R2: quarter-wave plate; L1, L2: lens; LED: light-emitting diode; CCD: charge-coupled device camera. The oblique incidence angle, *θ* is approximately 15° to avoid surface reflection from the skin sample. The diameter of the illumination area is approximately 1.8 cm. (**b**) Photograph of nude mouse skin sampling. A 1.5 cm × 1.5 cm square of back skin was chosen as the imaging region, as indicated by the red square.

The experimental setup adopted the typical dual rotating retarder configuration for backscattering Mueller matrix measurements^34–36^. As shown in Fig. 1(a), diverging light from the light-emitting diode (632 nm, 3 W, Cree, China) is collimated by a lens (L1, Thorlabs, USA). The polarization states of the incident light are controlled by a linear polarizer with a horizontal polarization direction (P1, extinction ratio >1000:1, Daheng Optic, China) and a rotating quarter-wave plate (R1, Daheng Optic). Thus, incident light with different polarization states is directed onto the sample. The backscattered photons from the sample pass through another rotating quarter-wave plate (R2, Daheng Optic) and horizontal polarizer (P2, extinction ratio >1000:1, Daheng Optic), and are recorded by a CCD camera (QImaging 32–0122 A, 12 bit, Canada). To eliminate surface reflection from the sample, there is an oblique angle (*θ* = 15°) between the incident light and the axis of analysis. During each measurement, the two retarders (R1, R2) rotate thirty times in total at fixed rates of *ω*_1_ = 5*ω*_2_. After being calibrated by measuring the Mueller matrices of standard samples such as air and retarders, the maximum error of the whole system was approximately 0.01.

For accurate and convenient measurements, we chose a smooth and uniform area on the backs of 8–9-week-old nude mice (provided by Guangdong Medical Lab Animal Centre, Guangzhou, Guangdong, China) as the skin sample location, which is marked by the red square in Fig. 1(b). To induce self-repairable UV skin damage, nude mice were irradiated with UVR (0.05 J/cm^2^) for five minutes per day over three days. Exposure to 0.05 J/cm^2^ of UV irradiation damaged the skin of nude mice^37^. Irradiation with 0.05 J/cm^2^ of UV 3 times/week for 30 weeks induced melanomas in 27–33% of nude mice skin samples that could not be self-repaired^38^. The source of UV radiation was two UV-B F15T8 lamps (wavelength 290 nm, Funakoshi, Tokyo). The distance between the UV-B lamps and nude mouse skin was 30 cm. The irradiation system is controlled by an ordinary electrical switch. The power density at the sample surface area was approximately 170 μw/cm^2^. During this irradiation, the microstructures of the skin were damaged. After each UVR injury, we obtained the backscattering Mueller matrices of the samples and quantitatively examined the relationship between the MMT parameters and the microstructural changes to the skin. Similarly, during the subsequent self-repairing stage, the Mueller matrices of the same skin areas were measured at 24 hour intervals over three to four days, until the skin samples were nearly restored to their original states. In order to avoid experimental errors arising from movement of the skin during data acquisition, the nude mice were injected with chloral hydrate (10%) to ensure they remained stationary during the measurement. To identify the irradiated areas for analyses, ink squares were marked on the back skin of nude mice before the first Mueller matrix imaging. In addition, hematoxylin–eosin (H-E) stained tissue sections of the skin samples were prepared for histological observation. The use of the animals in this study was approved by the Administrative Committee on Animal Research of the Graduate School at Shenzhen, Tsinghua University. All experiments and methods were performed in accordance with the relevant guidelines and regulations.

### Mueller Matrix Elements and Mueller Matrix Transformation (MMT) Parameters of Skin Samples

**Figure 2:**
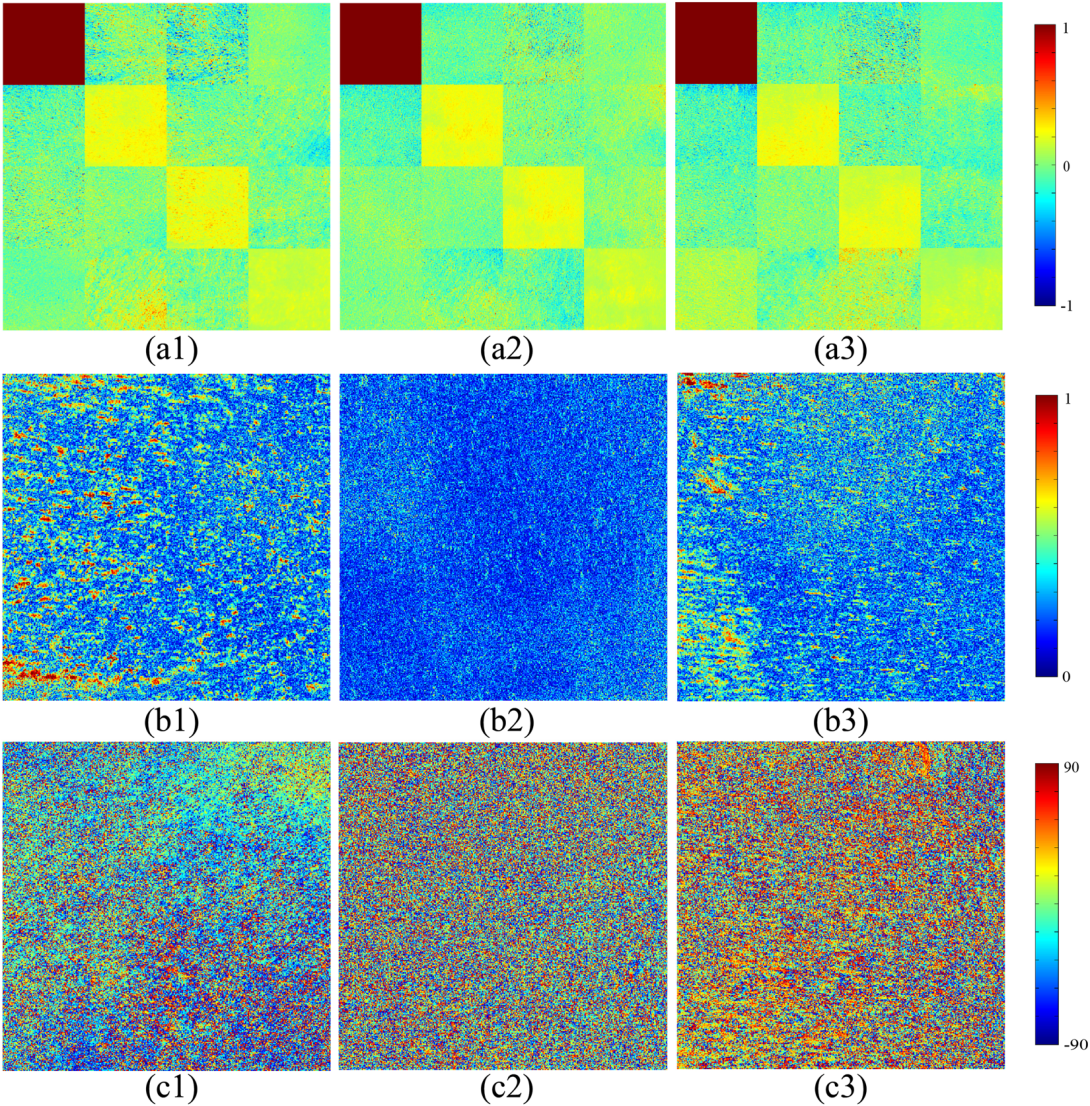
Mueller matrices and MMT parameters. (**a1**–**a3**) 2D images of backscattering Mueller matrices for a representative nude mouse skin sample during the photo-damaging and self-repairing processes: (**a1**) before UV damage; (**a2**) after the most serious UV damage; (**a3**) after the completion of self-repairing. All matrix elements are normalised by the m11 element. The colour scale represents normalised values from −1 to 1 for the m11, m22, m33, and m44 elements, and from −0.1 to 0.1 for the other elements. (**b1–b3**) 2D images of experimental MMT parameter *A* for the same nude mouse skin sample: (**b1**) before UV damage; (**b2**) after the most serious UV damage; (**b3**) after the completion of self-repairing. The colour scale represents values from 0 to 1. (**c1–c3**) 2D images of experimental MMT parameter *α* for the nude mouse skin sample: (**c1**) before UV damage; (**c2**) after the most serious UV damage; (**c3**) after the completion of self-repairing. The colour scale represents values from −90° to 90°. The size of the images is 0.8 × 0.8 cm^2^ (400 × 400 pixels).

Figure 2(a1–a3) shows the experimental 2D backscattering Mueller matrices of a nude mouse skin sample at different measurement times during the skin photo-damaging and self-repairing processes: before UV damage (Fig. 2(a1)), after the most serious UV damage, following 3 days’ UVR treatment (Fig. 2(a2)), and after self-repairing (Fig. 2(a3)). In our previous studies, we found that some characteristic structural features of bulk tissues can be obtained from the Mueller matrices. For instance, the magnitudes of the off-diagonal elements and the differences between the diagonal m22 and m33 elements are closely related to the anisotropy of tissues. Moreover, the orientation directions of the fibrous structures can be calculated by using the m22, m33, m23 and m32 elements^19,39^. Based on these studies, two conclusions can be drawn from Fig. 2(a1–a3). Firstly, the difference between m22 and m33, together with the prominent off-diagonal elements, demonstrates that the normal nude mouse skin sample was anisotropic. However, it can be observed from Fig. 2(a2) that the difference between m22 and m33, and the magnitude of the off-diagonal elements, became very small after the most serious UV damage, which indicates that the degree of anisotropy was reduced during photo-damaging. Secondly, the magnitudes and signs of most elements vary from Fig. 2(a1) to Fig. 2(a3), indicating that the directions of the fibrous structures had changed during the skin photo-damaging and self-repairing processes.

To disentangle the microstructural information encoded in the 2D images of the Mueller matrix elements, we employed the MMT parameters, *A* and *α*, which describe the anisotropy and the orientation of fibres^24^. Figures 2(b1–b3) and (c1–c3) show 2D images of parameters *A* and *α* from a nude mouse skin sample at different measurement times. Figure 2(b2) clearly shows the smallest value of parameter *A*, compared with Fig. 2(b1) and (b3), which indicates that the degree of anisotropy decreases with the development of skin photo-damaging. The value of parameter *A* then recovered significantly with the gradual completion of the self-repairing process, as shown in Fig. 2(b3). On the other hand, the 2D patterns of *α* shown in Fig. 2(c2) (after the most serious UV damage) are more uniformly and discretely distributed compared with Fig. 2(c1) (before UV damage) and Fig. 2(c3) (after the completion of self-repairing). These results indicate that the distribution of parameter *α* becomes wider in the skin photo-damaging process, and then gradually returns to the initial state in the self-repairing stage.

### Quantitative Analysis of Samples with and without Sunscreen

**Figure 3:**
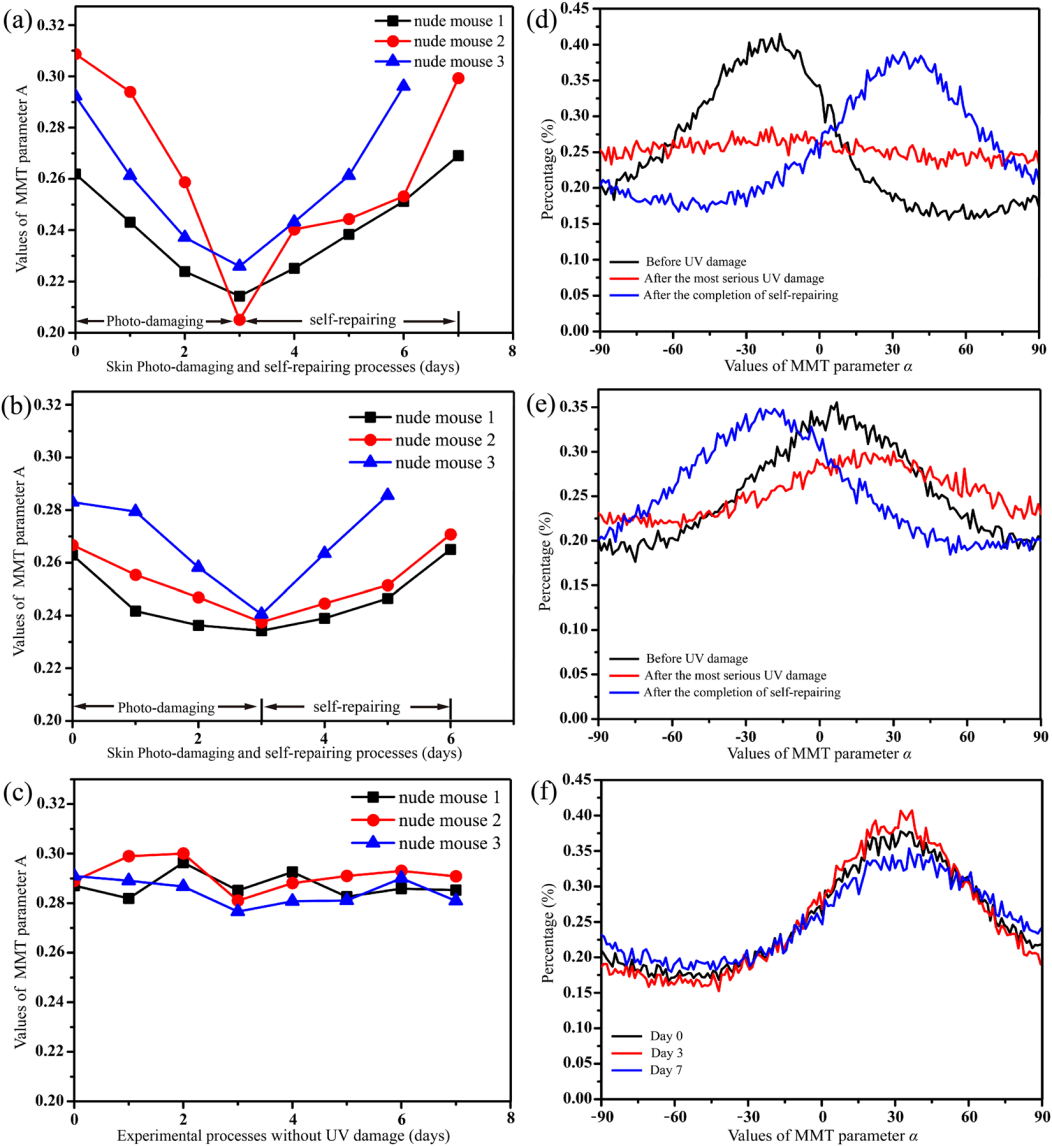
Skin samples with and without sunscreen. (**a–c**) Average values of MMT parameter *A* for three nude mouse skin samples (**a**) without and (**b**) with the application of sunscreen, or (**c**) not exposed to UV radiation. The horizontal axis represents the number of days from the beginning of the experiment. (**d–e**) FDH of MMT parameter *α* for a single nude mouse skin sample (**d**) without and (**e**) with sunscreen, at different measurement times: before UV damage (black line), after the most serious UV damage (red line), and after the completion of self-repairing (blue line). (**f**) FDH of MMT parameter *α* for a single nude mouse skin sample not exposed to UV radiation. The areas under the FDH curves are normalised to 1, and the horizontal axis is divided into 400 parts.

**Figure 4:**
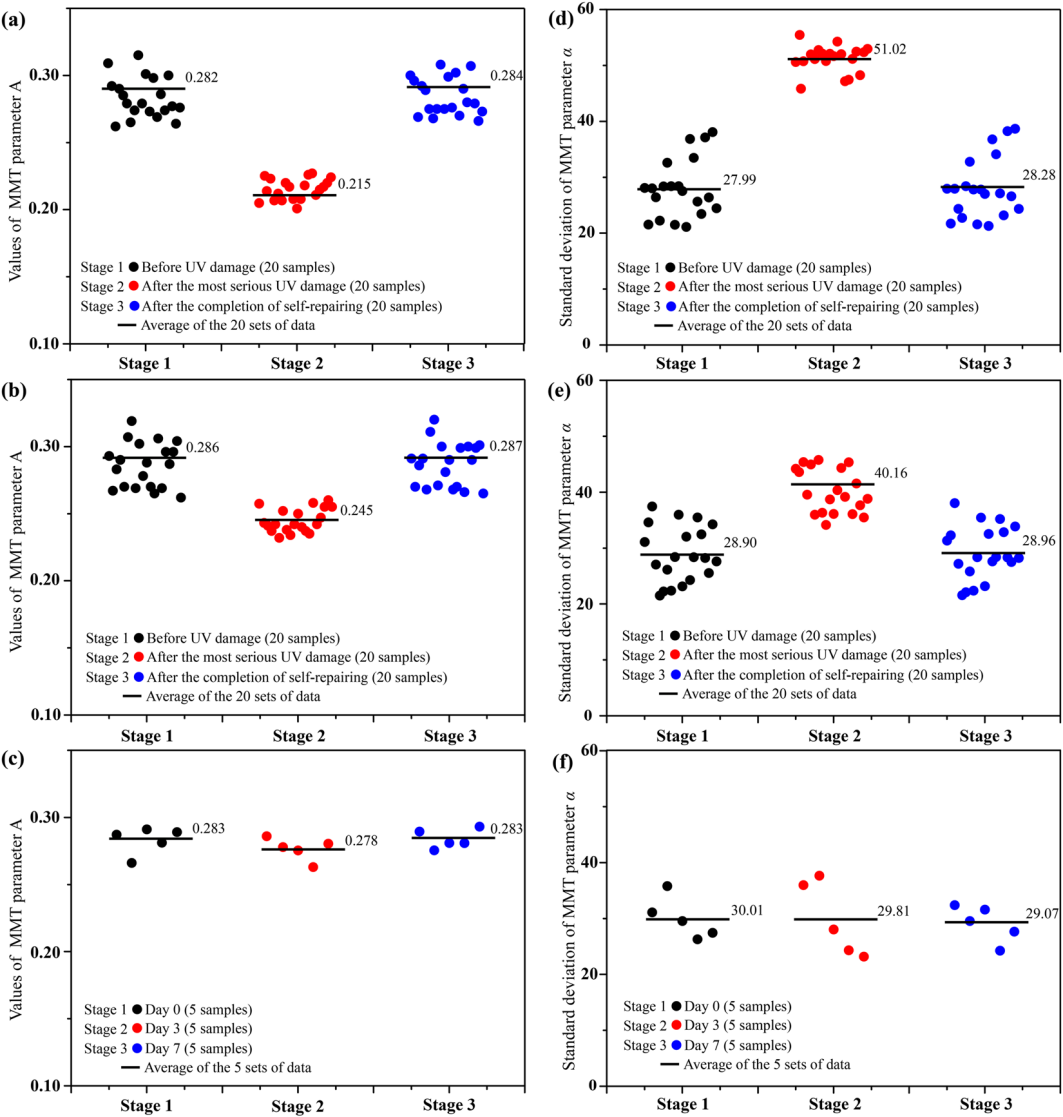
Skin samples with and without sunscreen. (**a** and **b**) Average values of MMT parameter *A* for twenty nude mouse skin samples (**a**) without and (**b**) with the application of sunscreen, at different stages: before UV damage (black dots), after the most serious UV damage (red dots), and after the completion of self-repairing (blue dots). (**c**) Average values of MMT parameter *A* for five nude mouse skin samples not exposed to UV radiation. (**d** and **e**) Standard deviations of MMT parameter *α* for twenty nude mouse skin samples (**d**) without and (**e**) with sunscreen, at different measurement times: before UV damage (black dots), after the most serious UV damage (red dots), and after the completion of self-repairing (blue dots). (**f**) Standard deviations of MMT parameter *α* for five nude mouse skin samples not exposed to UV radiation.

In this study, skin samples from 20 nude mice were analysed in a photo-damaging and self-repairing experiment. The MMT parameters *A* and *α* showed similar changes for all replicates. A more extensive quantitative analysis of three different mice that were not treated with sunscreen was undertaken using 400 × 400 pixels from each mouse. Our tests showed that using 400 × 400 pixels (0.8 × 0.8 cm^2^) for the back skin of small nude mice provided stable results for the analysis. As indicated in Fig. 3(a), the average values of parameter *A* for all three mice decreased monotonically as skin photo-damaging intensified in the first three days of UV damage. The values of parameter *A* then increased through the self-repairing stage until they recovered to their original state after three or four days. Moreover, as indicated in Fig. 4(a) and Supplementary Information Table S1, for all 20 nude mice in this study, the average values of parameter *A* were mostly between 0.26 and 0.32 before UV damage, were reduced to 0.20 to 0.23 after serious damage, and then increased to their initial magnitude after the completion of self-repairing. It can be observed from Fig. 4(a) that although the starting values of parameter *A* are different because of individual differences of nude mice, the photo-damaged and healthy skin samples can be distinguished clearly.

We found in our previous studies that the frequency distribution histograms (FDH) of the Mueller matrix parameters display the main structural features of samples in a clearer graphical form than the 2D images^40^. Thus, we transformed the 2D images of parameter *α* into a group of FDH curves to analyse changes in the fibrous structures of skin during the experimental process. The parameter *α* represents the orientation angle of the fibrous structures, which means that the standard deviation of its FDH is closely related to the degree of order of fibres: a larger standard deviation indicates more disordered fibrous structures. Figure 3(d) shows the FDH curves of parameter *α* for a nude mouse skin sample at three timepoints. It should be noted that the positions of the FDH peaks are not the same because the sample orientations varied between measurements. However, the distribution widths, which are independent of the peak positions, were used to determine the degree of fibre disorder. It is clear that, after the most serious UV damage, the distribution of parameter *α* (red line) becomes much wider compared with that before UV damage (black line) and after the completion of self-repairing (blue line). For quantitative analysis, we calculated the standard deviations of parameter *α* for all 20 nude mice, as shown in Fig. 4(d) and Supplementary Information Table S1. The mean standard deviations increased by approximately 23° after the most extensive UV damage (red dots, 45.44°–54.23°, mean value 51.02°) compared with undamaged skin (black dots, 21.14°–38.06°, mean value 27.99°). Furthermore, the standard deviations returned to their approximate initial values after the completion of self-repairing (blue dots, 21.33°–38.64°, mean value 28.28°). These results suggest that severe UVR may seriously damage the well-ordered fibres in normal skin tissues, causing the fibre orientation to become disordered. Subsequently, the fibrous structures rearrange during the process of self-repairing until they return to their original degree of order. The characteristic temporal patterns of variation in MMT parameters *A* and *α* were similar for the different mice, demonstrating that these parameters can be used as tools for quantitatively monitoring the structural changes of skin during the photo-damaging and self-repairing processes.

We next obtained Mueller matrices for 20 nude mouse skin samples that had been coated with sunscreen. The sunscreen’s main ingredients and their concentrations are avobenzone (3%), homosalate (8%), octisalate (5%), octocrylene (7%), oxybenzone (4%), zinc oxide (4%), titanium (IV) and oxide (6%). The UV blocking coefficient was SPF 50, PA+++. According to the instructions, the sunscreen was applied to a nude mouse 30 minutes before exposure to UV radiation. The quantity of sunscreen used in this study was 2 mg/cm^2^. Figure 3(b) and (e) show the average values of MMT parameter *A* for three samples, and FDH of parameter *α* for one sample at three timepoints. The reduction in the values of parameter *A* in the first three days was smaller after protection with sunscreen compared with the samples without sunscreen (Fig. 3(a)). Moreover, the standard deviation of parameter *α* increased to a lesser extent during the skin photo-damaging process, and the skin samples took less time to complete self-repairing (two or three days). As indicated in Fig. 4(b), (e) and Supplementary Information Table S1, the mean value of parameter *A* for all 20 nude mice coated with sunscreen in this study was decreased by 0.03 to 0.04 units in the photo-damaging stage. The standard deviation of parameter *α* increased by approximately 10° compared with undamaged skin, which is less than 50% of the change seen in samples without sunscreen.

To rule out any influence on the MMT parameters from natural variations of skin structure during the experiments, we also chose 5 nude mice for a control experiment. Figures 3(c),(f), 4(c),(f) and Supplementary Information Table S2 show that the values of *A* and standard deviations of *α* were relatively stable for nude mice not exposed to UVR. These data indicate that the sunscreen had a UVR protective effect on the skin, which was quantitatively evaluated by a non-contact polarization imaging technique. The preliminary experimental results confirm that the correct usage of SPF 50 PA+++ sunscreen can markedly reduce photo-damage, in accordance with our expectations. However, to determine a quantitative relationship between the reduction in the magnitude of photo-damage and the usage of sunscreens with different SPF values, further systematic analyses involving different experimental conditions should be carried out in future studies.

### Microstructural Changes of Skin Samples and MC Simulations

**Figure 5:**
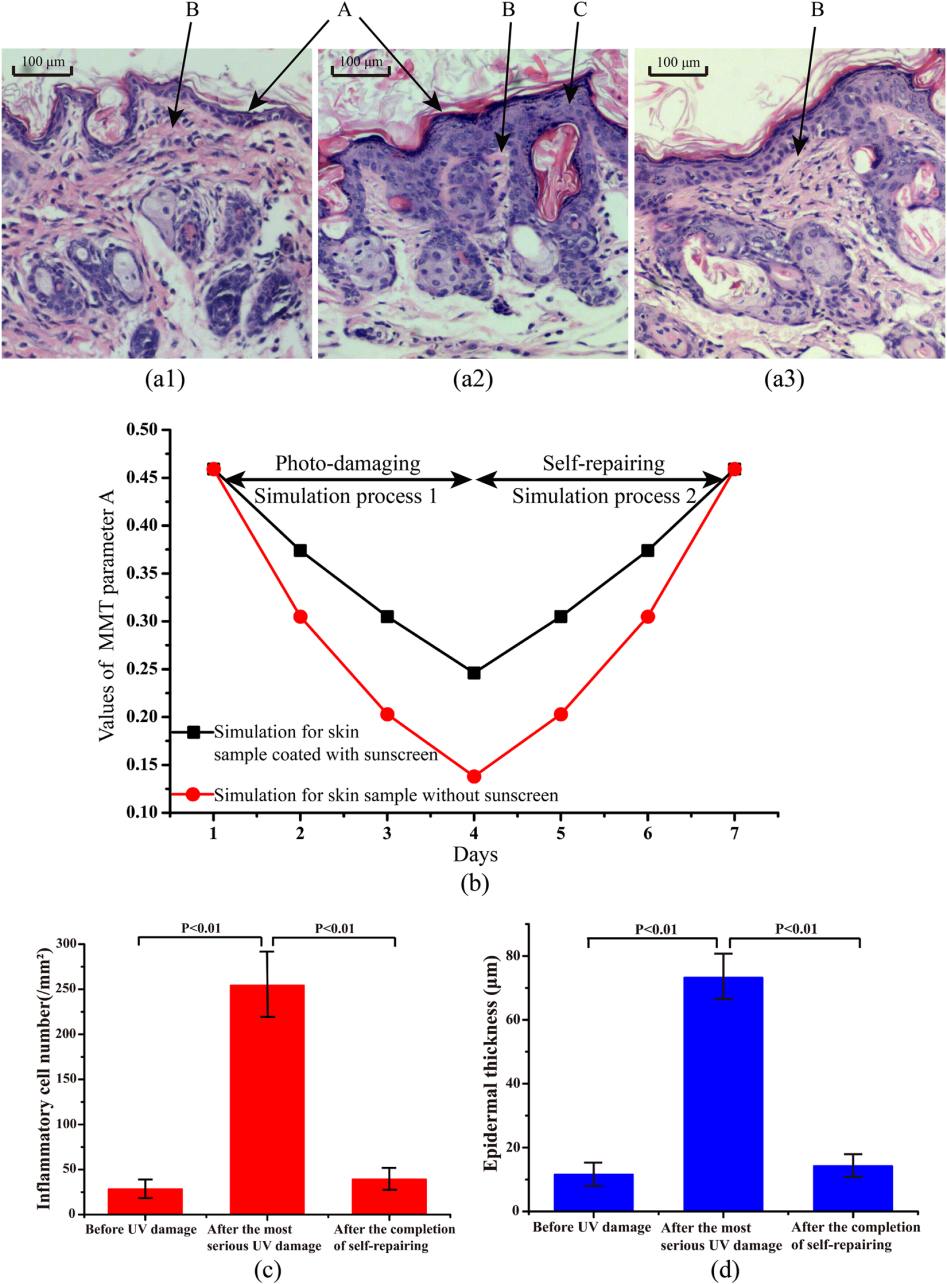
Skin histology and MC simulations. (**a1**–**a3**) Microscopic images of H-E stained sections of nude mouse skin (**a1**) before UV damage, (**a2**) after the most serious UV damage, and (**a3**) after the completion of self-repairing. Arrows show the skin surface (A), collagen fibres (B) and inflammatory cells (C). (**b**) Results of MC simulation for parameter *A* for nude mouse skin without sunscreen (red circles) and skin coated with sunscreen (black squares). For skin without sunscreen, the standard deviations of the angular distribution of the cylinders were (1) 10, (2) 14, (3) 18 and (4) 22. The values of the sphere-cylinder ratios were (1) 20:180, (2) 30:170, (3) 40:160 and (4) 50:150. For skin coated with sunscreen, the standard deviations of the angular distribution of the cylinders were (1) 10, (2) 12, (3) 14 and (4) 16. The values of sphere-cylinder ratios were (1) 20:180, (2) 25:175, (3) 30:170 and (4) 35:165. (**c** and **d**) Quantitative histological examination results for 18 nude mouse skin samples (**c**), mean number of inflammatory cells, and (**d**) mean epidermal thickness, at different stages. The histology parameters were determined visually from histological images by experienced pathologists.

To examine microstructural changes, we prepared H-E stained sections of nude mouse skin before UV damage, after the most serious UV damage, and after the completion of self-repairing (Fig. 5(a1–a3)). In the UV damaged region shown in Fig. 5(a2), the fibres appear disintegrated, inflammatory cells have increased near the skin surface where the UV damage is most severe, and the stratum corneum is thickened as a result of the UV damage response. In contrast, skin before UV damage (Fig. 5(a1)) and after the completion of self-repairing (Fig. 5(a3)) showed a large number of fibres and few inflammatory cells. Thus, the results of the Mueller matrix analyses above were confirmed by the histological data, whereby the reduction of fibres and the increase of inflammatory cells were evident in the most extensively UV damaged samples.

Clinically, UVR-induced damage is usually diagnosed by dermatoscope and allergy test. Dermatologists provide qualitative evaluations on skin conditions according to the results of these tests. For severe damage, further histological examinations should be carried out, which can provide quantitative diagnostic indicators such as the number of inflammatory cells, and epidermal thickness^41^. In this study, 18 mice were randomly chosen and sacrificed at different stages for histological examination (six mice before UV damage, six mice after UV damage, and six mice after self-repairing). As shown in Fig. 5(c) and (d), after UV damage, the mean number of inflammatory cells in nude mouse samples increased from 28/mm^2^ to 254/mm^2^, and the mean epidermal thickness increased from 12 μm to 74 μm. After self-repairing, the two indicators decreased to 38/mm^2^ and 15 μm, respectively. These quantitative histological examination results demonstrate the potential of Mueller matrix polarimetry to characterise skin conditions.

We conducted MC simulations based on the SCBM for a more detailed interpretation of the experimental observations described above. Since self-repairing (simulation process 2) is an inverse process of UV damage (simulation process (1), we focused on an analysis of the simulated parameter *A* in the skin photo-damaging process. Our previous experimental and simulated results demonstrated that the anisotropy of tissues may originate from both the optical birefringence and cylindrical scatterers, which can be distinguished by features in different Mueller matrix elements^19^. The experimental results above show that the changes seen in this study mainly affected the fibrous structures. Therefore, the parameters for the MC simulation were set as follows. The birefringence value, *Δn* was set to be 0.0001. The thickness of the medium was 0.05 cm. The refractive indices of the scatterers and interstitial medium were 1.4 and 1.33 respectively. To mimic the fibrous structures, nuclei and organelles in nude mouse skin, the diameters of the cylindrical and spherical scatterers were set at 1.5 µm and 0.2 µm^30^.

It has been reported in some publications that, during skin photo-damaging, UVR enhances the effect of hyaluronidase and elastase, leading to the degeneration of elastic fibres, the substantial reduction of collagen fibres and the disappearance of matrix in skin tissues^42,43^. Our previous study showed that the scattering coefficient of nude mouse skin was approximately 200 cm^−1^ ^44^. It also indicated that the process of fibres degeneration in tissues can be approximately described by a decrease in both the density and degree of order of fibrous structures^18^. Figure 5(a1–a2) shows that during the UV damage process, inflammatory cells were increased, the fibres disintegrated, and the stratum corneum thickened. Of note, during the photo-damaging and self-repairing processes, the scattering coefficient of the tissue will also change along with the distribution of spheres and cylinders. However, compared with the sphere-cylinder ratio, the change of the scattering coefficient is relatively small and parameters *A* and *α* are mainly affected by the sphere-cylinder ratio and the orientation distribution of cylinders. Thus, to simplify the MC simulation of anisotropic tissues, in our previous and current studies^18,28^, we maintained the total scattering coefficient constant and increased the standard deviation of the angular distribution of cylindrical scatterers and the sphere-cylinder ratio to simulate the decrease in degree of order and density of fibres in skin microstructures. Because photo-damage of the samples without sunscreen was more extensive than for samples coated with sunscreen, we adjusted the simulation parameters of the skin samples without sunscreen over a wider range. For skin samples without sunscreen, we varied the sphere-cylinder ratio from 20:180 to 30:170, 40:160 and 50:150; meanwhile, the standard deviation of the cylinders’ angular distribution was increased from 10° to 22° in 4° steps. For skin samples coated with sunscreen, the sphere-cylinder ratio was varied from 20:180 to 25:175, 30:170 and 35:165, and the standard deviation of the cylinders’ angular distribution was increased from 10° to 16° in 2° steps. The standard deviation of the angular distribution of cylindrical scatterers and the sphere-cylinder ratio values used in this study were determined according to experimental observations and suggestions from pathologists. As shown in Fig. 5(b), the MC simulated parameter *A* for skin samples without sunscreen decreased more as the UV damage developed than that for skin samples coated with sunscreen. The good agreement with the experimental results confirms that Mueller matrix derived parameters can non-invasively provide monitoring indicators for quantitatively characterising the microstructural features of skin tissues.

## Discussion

At present, the detection of skin structural variations is usually based on histological analysis of skin biopsies observed by optical microscopy^45^. However, this method cannot be performed in real time, is invasive, and needs stained tissue sections and experienced pathologists to provide accurate evaluation. Furthermore, removing skin tissue from its original environment may cause changes in its mechanical properties that affect the results. In daily life, skin tissue is vulnerable to UVR^46^. Many methods have been developed to protect the skin from UVR damage, including the use of various kinds of sunscreens. It would be helpful in the fields of both cosmetics and dermatological health to have a non-contact and low-cost technique for determining the degree of UVR damage and the effects of sunscreens in real time.

In this paper, we used MMT parameters *A* and *α* to characterise the microstructural changes of nude mouse skin during the photo-damaging and self-repairing processes. The reproducible changes in parameters *A* and *α* demonstrate that they can be used as tools for quantitatively monitoring structural changes in skin tissues. The comparisons between nude mouse skin coated with and without a sunscreen demonstrate that these parameters have the potential to evaluate the protective effect of sunscreens. According to the microscopic images of the H-E stained slices of nude mouse skin tissues, the contrast mechanism of the experimental results is backed up by MC simulations based on the SCBM. The consistency of the experimental and simulated data suggests that this technique may have good prospects for application in the fields of cosmetics and dermatological health.

## Methods

### Mueller Matrix Transformation (MMT) Parameters

As a comprehensive description of polarization-related structural information and the optical properties of a sample, the Mueller matrix can be used as a tool for tissue characterisation and biomedical research^47–49^. However, the Mueller matrix elements lack explicit association with the characteristic microstructural properties of samples and are often seriously affected by the orientations of fibrous structures^19,20^. Therefore, there are prominent difficulties in the practical biomedical application of Mueller matrix polarimetry. To overcome this problem, several methods have been proposed to transform Mueller matrix elements into parameters with clearer physical and structural meaning. For example, the Mueller matrix polar decomposition (MMPD) method proposed by Lu and Chipman decomposes a Mueller matrix into a set of sub-matrices representing a depolarizer, a retarder, and a diattenuator, respectively^23^. After the decomposition, the three sub-matrices can be transformed into specific parameters related to the depolarization, retardance, and diattenuation properties. In our previous studies, a set of orientation-insensitive polarization parameters were extracted using the MMT method to address this problem^24^. Compared with the MMPD parameters obtained from a decomposition process of all 16 Mueller matrix elements, each MMT parameter was calculated using a group of Mueller matrix elements. Therefore, the calculation of the MMT parameters was relatively faster than for the MMPD parameters^30^. In addition, the MMT parameters *A* and *α* used in this study were obtained from 4 linear polarization state related elements, indicating that when retarders are not required, the method can be easily combined with smart phones or wearable devices for daily skin monitoring.

1 $$\begin{array}{rcl}A & = & \frac{2\cdot \frac{m22+m33}{2}\cdot \frac{\sqrt{{(m22-m33)}^{2}+{(m23+m32)}^{2}}}{2}}{{(\frac{m22+m33}{2})}^{2}+{(\frac{\sqrt{{(m22-m33)}^{2}+{(m23+m32)}^{2}}}{2})}^{2}}\in [0,1]\\ tan\,\alpha  & = & tan(4x)=\frac{m23+m32}{m22-m33}\end{array}$$

Equation (1) defines the two MMT parameters used in this study. Considering that mouse skin tissues are highly depolarizing, these two parameters were chosen to give more precise measurements; they are extracted from the m22, m23, m32 and m33 elements, which have larger amplitudes compared with the other elements^24^. The parameters, *A* and *α* are independent, and our previous studies have demonstrated that they have different physical meanings: parameter *A* is closely related to the degree of anisotropy, or the order of alignment of the fibrous structures, while parameter *α* represents the orientation angle of the fibres^24^.

### Monte Carlo (MC) Simulation

For a better understanding of the relationship between skin microstructure and the polarization parameters, MC simulations based on SCBM were adopted to study the trajectories and polarization states of scattered photons as they propagate in nude mouse skin^31–33^. In this model, spherical scatterers with different sizes and infinitely long cylindrical scatterers are used to approximate the organelles and fibrous microstructures respectively. To mimic the skin tissues, the interstitial medium is birefringent. In this study, the parameters of the SCBM were set according to the characteristic features of the nude mouse skin samples observed during the photo-damaging and self-repairing processes.

### Data Availability

The datasets generated and analysed during the current study are available from the corresponding author on reasonable request.

## Electronic supplementary material

Supplementary information
