# Supplementary material for: A quantitative and non-contact technique to characterise microstructural variations of skin tissues during photo-damaging process based on Mueller matrix polarimetry
Source: Sci Rep. 2017 Oct 31;7:14702. doi: 10.1038/s41598-017-14804-z (PMC5666003; doi:10.1038/s41598-017-14804-z)
Supplement: Supplementary file 1 — Supplementary information [file 41598_2017_14804_MOESM1_ESM.pdf]

A quantitative and non-contact technique to characterise microstructural variations of skin tissues during photo-damaging process based on Mueller matrix polarimetry

Yang Dong, Honghui He, Wei Sheng, Jian Wu & Hui Ma

Table 1. Comparison of the MMT parameters, A and α between nude mouse skin samples without and with the application of sunscreen, during the skin photo-damaging and self-repairing processes

**Average values of MMT parameter *A***								
Skin samples without sunscreen* \ *Days	**Day 0**	**Day 1**	**Day 2**	**Day 3**	**Day 4**	**Day 5**	**Day 6**	**Day 7**
Sample 1	0.309	0.295	0.261	0.205	0.242	0.244	0.253	0.300
Sample 2	0.292	0.260	0.239	0.226	0.243	0.261	0.296	
Sample 3	0.262	0.242	0.229	0.214	0.231	0.229	0.254	0.269
Sample 4	0.290	0.268	0.242	0.223	0.234	0.255	0.273	0.292
Sample 5	0.276	0.266	0.237	0.207	0.229	0.257	0.273	
Sample 6	0.274	0.253	0.256	0.212	0.244	0.264	0.275	
Sample 7	0.265	0.249	0.239	0.207	0.221	0.224	0.251	0.268
Sample 8	0.300	0.277	0.244	0.220	0.221	0.252	0.287	0.307
Sample 9	0.315	0.290	0.251	0.217	0.234	0.267	0.289	0.308
Sample 10	0.279	0.257	0.254	0.208	0.225	0.253	0.275	
Sample 11	0.274	0.242	0.239	0.201	0.221	0.238	0.259	0.280
Sample 12	0.298	0.264	0.235	0.208	0.215	0.224	0.267	0.302
Sample 13	0.273	0.251	0.238	0.218	0.223	0.245	0.276	
Sample 14	0.269	0.268	0.238	0.226	0.242	0.254	0.270	
Sample 15	0.286	0.258	0.242	0.227	0.238	0.275	0.270	0.290
Sample 16	0.301	0.252	0.239	0.211	0.220	0.255	0.299	
Sample 17	0.274	0.267	0.248	0.215	0.239	0.266	0.275	
Sample 18	0.277	0.252	0.238	0.217	0.221	0.243	0.259	0.279
Sample 19	0.264	0.261	0.242	0.220	0.239	0.253	0.266	
Sample 20	0.285	0.263	0.246	0.224	0.234	0.253	0.271	0.289

Skin samples coated with sunscreen \ *Days*	**Day 0**	**Day 1**	**Day 2**	**Day 3**	**Day 4**	**Day 5**	**Day 6**	**Day 7**
Sample 1	0.262	0.243	0.239	0.234	0.241	0.250	0.265	
Sample 2	0.267	0.258	0.247	0.238	0.245	0.255	0.270	
Sample 3	0.283	0.280	0.259	0.241	0.270	0.286		
Sample 4	0.290	0.287	0.263	0.242	0.246	0.273	0.291	
Sample 5	0.296	0.277	0.254	0.242	0.269	0.300		
Sample 6	0.307	0.283	0.270	0.258	0.278	0.311		
Sample 7	0.319	0.302	0.280	0.252	0.272	0.269	0.320	
Sample 8	0.306	0.287	0.253	0.243	0.278	0.299		
Sample 9	0.302	0.298	0.276	0.259	0.255	0.281	0.300	
Sample 10	0.278	0.267	0.252	0.242	0.262	0.281		
Sample 11	0.288	0.278	0.266	0.250	0.272	0.290		
Sample 12	0.270	0.259	0.245	0.240	0.252	0.268		
Sample 13	0.265	0.252	0.241	0.237	0.244	0.251	0.270	
Sample 14	0.269	0.260	0.241	0.235	0.235	0.271		
Sample 15	0.269	0.249	0.250	0.235	0.245	0.266		
Sample 16	0.270	0.263	0.252	0.237	0.249	0.268		
Sample 17	0.287	0.266	0.260	0.247	0.255	0.271	0.290	
Sample 18	0.296	0.286	0.268	0.255	0.271	0.285	0.299	
Sample 19	0.304	0.294	0.284	0.260	0.262	0.280	0.301	
Sample 20	0.293	0.290	0.272	0.255	0.261	0.277	0.291	
**Standard deviation of MMT parameter *α***								
Skin samples without sunscreen \ *Days*	**Day 0**	**Day 1**	**Day 2**	**Day 3**	**Day 4**	**Day 5**	**Day 6**	**Day 7**
Sample 1	21.54	24.05	32.69	51.43	40.00	42.01	36.92	21.73
Sample 2	28.08	36.97	45.64	50.58	44.67	37.30	28.21	27.95
Sample 3	28.01	33.77	42.26	50.72	43.95	36.62	32.01	27.48
Sample 4	26.44	31.99	43.10	45.44	44.67	36.04	30.70	27.55
Sample 5	28.40	31.95	42.34	52.05	47.52	35.90	27.82	
Sample 6	28.37	35.06	41.83	51.10	39.91	32.31	28.41	
Sample 7	32.57	35.95	39.06	52.72	43.95	40.14	39.54	32.75
Sample 8	22.27	31.60	39.99	51.95	50.80	42.02	31.59	22.75
Sample 9	21.50	23.15	38.22	51.95	43.09	32.36	38.17	21.60
Sample 10	28.40	34.16	37.15	52.05	46.31	37.81	31.26	
Sample 11	27.56	38.71	41.36	51.65	46.03	43.54	35.88	27.02
Sample 12	21.14	29.56	39.52	54.23	50.74	47.91	36.38	21.33
Sample 13	36.84	34.70	40.35	52.01	48.35	42.62	36.77	

Sample 14	33.47	34.18	38.72	51.15	42.97	35.82	33.08	
Sample 15	25.68	34.26	36.93	47.44	40.21	37.15	38.20	27.15
Sample 16	23.47	37.29	43.05	47.95	44.30	36.03	23.19	
Sample 17	37.10	41.22	51.10	52.45	47.04	42.61	38.22	
Sample 18	26.40	32.39	43.54	52.93	51.07	44.36	41.42	26.62
Sample 19	38.06	38.80	47.48	52.36	47.95	42.93	38.64	
Sample 20	24.48	31.11	41.96	48.24	47.55	40.25	30.25	24.35
Skin samples coated with sunscreen* / *Days	**Day 0**	**Day 1**	**Day 2**	**Day 3**	**Day 4**	**Day 5**	**Day 6**	**Day 7**
Sample 1	31.07	40.36	43.96	44.16	40.48	35.52	31.30	
Sample 2	34.58	36.04	40.67	43.75	39.95	36.60	32.26	
Sample 3	37.42	36.11	42.69	45.36	43.92	38.02		
Sample 4	27.06	28.51	33.86	39.52	35.99	31.77	27.20	
Sample 5	26.16	30.23	35.14	39.14	32.36	25.84		
Sample 6	22.26	27.92	32.76	35.96	30.42	22.13		
Sample 7	21.35	23.92	27.37	36.06	31.57	24.34	21.60	
Sample 8	22.41	27.60	35.90	36.31	31.10	22.38		
Sample 9	23.16	26.12	29.59	34.12	34.44	24.95	23.22	
Sample 10	28.37	34.38	35.96	38.69	35.95	28.42		
Sample 11	28.40	32.81	33.20	36.09	32.77	28.38		
Sample 12	32.02	36.04	38.17	40.36	38.17	32.50		
Sample 13	34.22	37.80	39.99	44.30	43.45	37.56	33.83	
Sample 14	35.94	36.00	40.48	44.92	47.49	35.41		
Sample 15	35.46	40.36	40.31	45.33	40.78	35.20		
Sample 16	32.45	36.17	40.48	45.76	40.96	32.82		
Sample 17	28.22	34.17	34.62	41.52	39.90	32.05	28.32	
Sample 18	25.55	28.52	35.99	37.65	36.26	29.76	26.52	
Sample 19	24.29	27.96	28.52	35.45	35.40	30.61	25.64	
Sample 20	27.64	30.00	32.44	38.79	35.02	31.86	28.22	

Table 2. MMT parameters A and α of nude mouse skin samples not exposed to UV radiation

**Average values of MMT parameter *A***								
Skin samples without UV damage* / *Days	**Day 0**	**Day 1**	**Day 2**	**Day 3**	**Day 4**	**Day 5**	**Day 6**	**Day 7**
Sample 1	0.287	0.282	0.296	0.285	0.293	0.283	0.286	0.285
Sample 2	0.266	0.263	0.282	0.279	0.269	0.271	0.281	0.277
Sample 3	0.291	0.289	0.287	0.277	0.281	0.281	0.290	0.281

Sample 4	0.281	0.272	0.268	0.269	0.279	0.282	0.278	0.281
Sample 5	0.289	0.299	0.300	0.281	0.288	0.291	0.293	0.291
**Standard deviation of MMT parameter α**								
Sample 1	31.07	32.02	30.72	35.94	33.50	36.04	31.07	32.45
Sample 2	35.78	31.60	32.71	37.64	34.04	29.15	35.78	29.51
Sample 3	29.51	27.14	32.35	28.02	32.96	26.22	29.51	31.55
Sample 4	26.26	26.95	23.74	24.29	24.25	26.12	26.26	24.22
Sample 5	27.42	25.63	26.51	23.16	25.10	23.32	27.42	27.64
